# Output-Only Damage Detection of Shear Building Structures Using an Autoregressive Model-Enhanced Optimal Subpattern Assignment Metric

**DOI:** 10.3390/s20072050

**Published:** 2020-04-06

**Authors:** Liu Mei, Huaguan Li, Yunlai Zhou, Dawang Li, Wujian Long, Feng Xing

**Affiliations:** 1 Guangdong Provincial Key Laboratory of Durability for Marine Civil Engineering, Shenzhen Durability Center for Civil Engineering, College of Civil and Transportation Engineering, Shenzhen University, Shenzhen 518060, China; meiliu@szu.edu.cn (L.M.); lihuaguan2017@email.szu.edu.cn (H.L.); lidw@szu.edu.cn (D.L.); xingf@szu.edu.cn (F.X.); 2 Department of Civil and Environmental Engineering, The Hong Kong Polytechnic University, Hong Kong 999077, China

**Keywords:** damage indicator, autoregressive model, optimal subpattern assignment distance, hungarian algorithm, multi-target tracking, shake table test

## Abstract

This paper proposes a novel output-only structural damage indicator by incorporating the pole-based optimal subpattern assignment distance with autoregressive models to localize and relatively assess the severity of damages for sheared structures. Autoregressive models can model dynamic systems well, while their model poles can represent the state of the dynamic systems. Structural damage generally causes changes in the dynamic characteristics (especially the natural frequency, mode shapes and damping ratio) of structures. Since the poles of the autoregressive models can solve the modal parameters of the structure, the poles have a close relationship with the modal parameters so that the changes in the poles of its autoregressive model reflect structural damages. Therefore, we can identify the damage by tracking the shifts in the dynamic system poles. The optimal subpattern assignment distance, which is the performance evaluator in multi-target tracking algorithms to measure the metric between true and estimated tracks, enables the construction of damage sensitive indicator from system poles using the Hungarian algorithm. The proposed approach has been validated with a five-story shear-building using numerical simulations and experimental verifications, which are subjected to excitations of white noise, El Centro earthquake and sinusoidal wave with frequencies sweeping, respectively; the results indicate that this approach can localize and quantify structural damages effectively in an output-only and data-driven way.

## 1. Introduction

Structural damage detection, an essential component of structural health monitoring (SHM), serves as a necessary measure for aging infrastructures during their long-term service to assess operating conditions and provide suggestions for potential repair and maintenance actions [1]. Over the past decades, various non-destructive testing (NDT) techniques have been developed for damage detection in structures such as magnetic particle testing, X-ray testing, ultrasonic testing, and so on [2]. In recent studies, NDT technologies have been widely used and proven quite effective in inspection of damage in composite structures. The latest research on these technologies includes eddy current testing of manufacturing flaws and operational damage [3,4,5], microwave nondestructive evaluation of galvanic corrosion and impact damage [6,7,8], and guided wave detection for various composite damage types [9,10,11]. All of these localized experimental methods can locate and identify the damage when the nearby region of the damage is known a priori. Besides, easy accessibility to the portion of the structure being scrutinized has always been a prerequisite. Bound by these restrictions, such NDT methods are primarily used for damage severity checks on or close to the surface of the structure. However, without stripping the architectural cladding at a high cost, it is difficult to evaluate the health condition of the inner components of the structure by using most standard NDT procedures. Thus, it is always expensive and difficult to use these local NDT methods to identify the damage for complex and large-scale structures without a priori knowledge of the possible damage sites. Generally, for complex and massive structures, some hybrid ways will most likely be employed by combining these local inspection methods with the quantitative global damage identification methods.

Among all global damage detection techniques, the vibration-based technologies occupy an essential role because of their characteristics of smooth conduction, high efficiency, and low cost. By using vibration-based methods, damage can be detected in a global sense even when the location of the damage is inaccessible. The basic idea of vibration-based damage detection methods is that the measured global dynamic properties, such as frequencies, mode shapes and modal damping, are functions of the physical properties of the structure (mass, stiffness and energy dissipation properties). Therefore, damage will alter the physical properties of a structure, which, in turn, will cause changes in the global dynamic properties. With a combination of local NDT techniques and global assessment, some hybrid approaches are considered as the most probable path for the practical applications. For instant, vibration-based techniques are used to narrow down the damage range to a certain portion of the structure. Then the local NDT methods are further used to locate and quantify damage on the structural components within the specific damaged part. 

Vibration-based damage identification methods have been a research hotspot in the past few decades due to their effectiveness and feasibility [12]. Previous studies adopted the changes of modal parameters between undamaged and damaged structures as the damage indices [13,14,15,16]. For damage identification, since the modal parameters represent global information of the overall structure, conventional modal-parameter based damage indicators rely on global structural damage and are usually not sensitive enough for local damage identification under complex environmental conditions [17,18,19]. On the other hand, in terms of SHM, nonlinearities that have resulted from the new composite materials’ properties, hysteretic behavior, complex geometry, and environmental conditions [20,21,22,23] restrict the further development of modal-parameter based approaches because of the difficulties in constructing complex finite element (FE) models with such nonlinearities. For instance, model-updating methods have to construct FE models precisely and inevitably induce huge computation load [24,25,26]. In such a case, the data-driven approaches become attractive in further investigation of the damages, especially for large-scale structures like long-span bridges and high-rise buildings.

Various approaches have been developed to deal with the challenges brought by modal-parameter based methods from both time domain such as blind source separation (BSS) [27,28], stochastic subspace identification (SSI) and system identification [29], and frequency domain such as frequency domain decomposition (FDD) and transmissibility [30]. For linear time-invariant (LTI) systems, simple mathematical black box models can interpret distinct system dynamics, enables the wide application, within which the AR model is typically illustrated in the discrete-time domain [31]. Besides, another advantage of the AR model is that the underlying process of generating observations can be inferred directly from the AR parameters without the need for spectral representation [32]. The use of AR parameters can characterize the structural features in order to address the drawbacks of conventional modal parameter based approaches in engineering application. Sohn and Farrar [33] constructed an autoregressive with exogenous inputs (AR-ARX) model utilizing its standard deviation of the residual as a structural damage indicator. Zheng and Mita [34] explored new applications of Itakura distance and cepstral distance measures for damage detection in civil engineering. Mahalanobis distance and Cosh spectral distance were introduced to the AR coefficients and AR model spectra to analyze the damages and noise sensitivity [35]. The AR model order influence on damage detection was discussed in [36]. Ma et al. [37] proposed a hybrid model based on the combination of a general expression for linear and nonlinear autoregressive (GNAR) model and a generalized autoregressive conditional heteroscedasticity (GARCH) model. They applied it to a rolling bearing fault diagnosis task based on the GNAR-GARCH model parameter identification. However, the previous time-domain approaches still have not fully resolved the difficulties encountered in real engineering applications. For instance, how to interpret the large amount of measured data, how to obtain the distinct severities of the real damages in complex structures, how to achieve the quantitative recognition rather than qualitative identification, and how to combine the damage indicators with practical SHM system are still challenging tasks especially for complex structures in engineering fields. 

To tackle the drawbacks of original time series-based methods, many improved time-domain approaches have been developed, leading to the following improvements in recent years: Xing and Mita [38] developed a substructure approach adopting the auto-regressive moving average with exogenous inputs (ARMAX) model to obtain the modal information of the substructures, which requires only three sensors to identify localized damage in any story of a shear structure building. Following this approach, Mei et al. [39] proposed an ARMAX model residual-based technique in order to improve the noise immunity and damage detection robustness by correcting the former damage indicator. An adaptive ARX model was constructed by Barraza et al. [40] to estimate the remaining useful life (RUL) for the aluminum crack growth problem. Besides, in order to solve the underdetermined problems caused by partial sensor input, some researchers have developed the damage detection methods in real-time using a subset of sensor inputs [41,42]. Hernandez [43] used an *l*_1_-norm minimization to identify the localized structural damage from incomplete spectrum information. He et al. [44] developed a damage diagnosis approach of a cracked beam based on mode shape reconstruction using the empirical mode decomposition (EMD) method with intermittency criteria. Ding et al. [45] proposed an improved Jaya (I-Jaya) algorithm using sparse regularization and Bayesian inference to detect structural damages under data with significant uncertainties and limited measurement information.

Additionally, to deal with the randomly high-dimensional damage-sensitive features under environmental and operational variability, Entezami et al. [46] proposed a feature extraction approach based on an AR model residual and a statistical distance method named partition-based Kullback Leibler Divergence (KLD) for damage detection. Zhou et al. [47] combined a vector time-dependent autoregressive (TAR) model with a least squares support vector machine to identify the structural parameters for linear time-varying structural systems. To improve assessment accuracy for early detection of faults in fixed-axis gearbox, Chen et al. [48] proposed a sparse functional pooled auto-regression (FP-AR) model using sparse AR terms and non-identical functional spaces.

Nevertheless, these newly developed time series modeling methods still have some shortcomings. Firstly, the previous ARX- or ARMAX-based methods normally require prior knowledge of structural excitations. Still, due to the presence of unknown excitations or the imprecise measurement acquisition of excitations in practical engineering applications, it would unavoidably bring some challenges. Secondly, the existing methods for solving undetermined problems by using a subset of inputs normally require accurately known modal information such as natural frequencies or mode shape, which would pose extra challenges to the identification results that rely on precise modal identification. Thirdly, since the pattern recognition based damage indicators still need a mass of training data from various damage scenarios, it is hard for these methods to extract damage sensitive features and reflect the damages without enough training data. Meanwhile, it unavoidably requires high calculation cost and long calculation time, and their disadvantages are not beneficial to the real practical health monitoring system. 

Therefore, it is still necessary for researchers in this area to devote further efforts to the construction of more enhanced structural damage indicators. Due to the rapid development of technologies in various fields, especially machine learning and artificial intelligence, the authors believe that the combination of technologies in different fields is promising to provide new ideas for solving engineering problems. In recent years, the optimal subpattern assignment (OSPA) distance has been widely applied in the multi-target tracking [49,50,51,52,53,54] filed for accurate quantification of similarity between the true tracks and estimated tracks. OSPA was originally developed by Schuhmacher et al., who applied it to assess the performance of multi-target tracking algorithms [49]. A relatively small OSPA distance value represents an ideal result, indicating that the estimated tracks are closed to the true tracks. The OSPA metric is taken to discriminate between the semantic object class of interest and other look-alike classes, leading to a weakly supervised multi-object detector [50]. Due to its sensitivity and effectiveness, the OSPA distance has been utilized in numerous areas including target tracking [51], system identification [52], and objective detection [53], which shows the excellent availability of OSPA metric based platform for other practitioners. Chlebek and Hanebeck [54] proposed a pole-based OSPA distance measure for change detection of a linear dynamic system in the machine learning field, which provides latent possibilities of signal feature extraction for decision-making in engineering detections.

Mei et al. [55] firstly proposed a structural damage detection approach using pole-based OSPA distance as a damage indicator via a substructure division strategy combined with the ARMAX model. In this method, since ARMAX modelling for each substructure is required, there is a need to capture the input information of the structure. However, in many engineering fields, such measurement of structural input information has proven difficult [56]. In turn, the inaccurate measurement of structural input information will cause the spurious damage detection results. In this case, using the output-only damage detection method without complete prior excitation knowledge becomes an effective and alternative way [57,58,59,60].

Furthermore, this method requires constructing a dynamic model of the substructure in advance, and the dynamic behavior of the actual structure will be more complicated, which will challenge the accuracy of the theoretical dynamic model in practical application. On the other hand, since the real structures in most engineering fields are subjected to multiple loads, the complex structural inputs make the construction of ARMAX model more complicated, which significantly reduces the calculation flexibility. Therefore, the previous damage detection approach [55] still needs to be improved to a more flexible and feasible version.

For the sake of addressing the limits of existing methods discussed above, this study explores an innovative application of OSPA distance measure for localized structural damage detection in an output-only way by constructing a new damage indicator. Compared to the previous method, the proposed damage indicator mainly devotes efforts to the following contributions: (1) the proposed damage indicator is flexibly calculated in an output-only way, requiring no prior knowledge about the structural excitation or structural input information; (2) the proposed method does not need to establish a substructural dynamic model in advance, avoiding the damage detection results to rely on the high accuracy of the complex physical modelling process; (3) unlike the previous ARMAX model based OSPA metric constrained by the structural inputs, the output-only AR-enhanced OSPA metric insures that the complexity of the calculation model will not be significantly increased by the complicated structural inputs, thus greatly increasing the flexibility of the calculation process. In this study, damage detection processes are intuitively achieved by the target tracking of AR poles. The AR poles of undamaged system are regarded as the true tracks while AR poles of damaged system are compared to the estimated tracks. Thus the change of poles between undamaged system and damaged system contains essential structural information of damage location and severity. AR poles are extracted from each story response, representing the localized structural property to achieve damage localization. It is different from those modal parameters based damage indicators, which can only reflect the overall structural damage. The magnitude of pole-based OSPA distance is able to quantify the difference between undamaged structure and damaged structure, clearly indicating the severity of structural damage. Furthermore, after defining pole-based OSPA distance between AR models, a procedure is developed for localizing and quantifying the structural damage in a data-driven way: firstly, a decorrelation procedure is adopted to remove the spatial correlation of structural responses on distinct sites due to the subsistent correlation of excitations in practical civil engineering structures; secondly, AR models of both undamaged and damaged structures are constructed through their time-series based responses; subsequently, poles of AR models are obtained by AR coefficients estimated from the structural responses; finally, the pole-based OSPA distance between AR models of undamaged and damaged structure, is calculated by Hungarian algorithm [61] from operational research to detect damages.

## 2. Theoretical Background

### 2.1. Dynamic System Modeling Using AR Model

In structural dynamic modeling analysis, the linear discrete-time system can be interpreted by the autoregressive process [32]:

(1) $$x\left(t\right)+\overset{q}{\underset{i=1}{\sum }}a_{i}x\left(t-i\right)=e\left(t\right)$$

where x(t) represents the output of the system at time *t*, e(t) indicates the white noise sequence of zero mean and variance $\sigma^{2}$, *i* is the counting variable, q denotes the model orders of the AR process, $a_{i}$ means the AR coefficient. The *z*-domain transfer function is hereinafter expressed as:

(2) $$H\left(z\right)=\frac{1}{A\left(z\right)}=\frac{1}{1+\sum_{i=1}^{q}a_{i}z^{-i}}$$

where *z* denotes the independent variable in the z domain, H(z) depicts the system function in the z domain, A(z) indicates the denominator polynomial of transfer function. The AR coefficients can be estimated by the well-known Burg’s method [62] based on forward and backward prediction errors; complex poles, which are denoted as $\left\{\alpha_{1},\alpha_{2},\ldots ,\alpha_{q}\right\}$, satisfy the condition for which H(z)=∞ and are determined by the AR coefficients $\left\{a_{1},a_{2},\ldots ,a_{q}\right\}$, i.e., the roots of the polynomial A(z); model order can be given by the extensively Akaike Information Criterion (AIC) [63], which is defined by:

(3) $$AIC(q)=N\ln\hat{\sigma }q2+2q$$

where *q* depicts the model order, *N* represents the number of observations to which the model is fitted, and ${\hat{\sigma }}_{q}^{2}$ denotes the estimated variance of the linear prediction error.

### 2.2. Optimal Subpattern Assignment Metric

The spatially finite sets of targets are called tracks, which involve target position or speed in Cartesian space, and may also consist of certain target features such as amplitude, size and shape. Compared with the error between single-target systems, the OSPA distance considers more information about the optimal permutation and combination of the subsets of tracks. It allows the inequality of the number of subsets between the true tracks and estimated tracks, which addresses the issues of an unknown number of targets and the uncertainty of target position in the real multi-target tracking system. The smaller the OSPA values, the closer the estimate tracks are to the true tracks. For more detailed knowledge of multi-target tracking, the readers can refer to [49,50,51,52,53,54]. In multi-target tracking, the set of true tracks ***X*** and the set of estimated tracks ***Y*** without labeled sequences are defined as:

(4) $$\{\begin{array}{c} X=\left\{x_{1},\ldots ,x_{i},\ldots ,x_{m}\right\}\\ Y=\left\{y_{1},\ldots ,y_{i},\ldots ,y_{n}\right\} \end{array}$$

where ***X*** and ***Y*** are arbitrary finite sets, the subsets $x_{i}\in {ℜ}^{N}$ and $y_{i}\in {ℜ}^{N}$ are *N*-dimension state vectors, *m* and *n* denote the numbers of subsets of X and Y, respectively. 

For the case m≤n, the original OSPA metric [49] between the set X of true tracks and the set Y of estimated tracks is defined as:

(5) $${\bar{d}}_{p}^{\left(c\right)}\left(X,Y\right)={\left(\frac{1}{n}\left(\underset{\pi \epsilon \prod n}{\min}\overset{m}{\underset{i=1}{\sum }}{\left(d^{\left(c\right)}\left(x_{i},y_{\pi \left(i\right)}\right)\right)}^{p}+c^{p}\left(n-m\right)\right)\right)}^{1/p}$$

where 1≤p<∞ depicts the metric order; the cut-off distance between arbitrary subset x and arbitrary subset y is defined as:

(6) $$d^{\left(c\right)}=min\left\{c,d\left(x,y\right)\right\}$$

where *c* > 0 indicates a cut-off parameter representing the minimal cut-off distance, d(x,y) depicts the base distance between two tracks, which could be Euclidean distance or Mahalanobis distance; ∏n represents the set of all permutations of length *m* with elements taken from {1,2,…,n}; $y_{\pi \left(i\right)}$ means the *i*th subset of the estimated tracks Y under the permutation case ‘π’; if m>n, ${\bar{d}}_{p}^{\left(c\right)}\left(X,Y\right)={\bar{d}}_{p}^{\left(c\right)}\left(Y,X\right)$. In terms of the relation with multi-target tracking, a dynamic system is similar to a tracking target in the state space, and the system parameters characterizing the dynamic system are compared to the corresponding tracks of the target. Initially, parameters of the system in undamaged state can be regarded as the true tracks. Once this undamaged system suffers from system change, such as stiffness degradation in engineering structures, it is reasonable to re-estimate the system parameters that are regarded as the estimated tracks. In order to quantify the difference between the undamaged system and the unknown system, parameters-based OSPA distance considering various system parameters and its all-permutations can give a comprehensive quantification for system comparing. In this way, the damage detection process is transformed into a target-tracking problem of system parameters. According to the calculated magnitudes, the closer the OSPA value is to zero, the closer the system state is to the undamaged state. Conversely, a large OSPA value indicates more severe damage existing in the system. In this study, the true tracks in multi-target tracking are replaced with AR poles in the undamaged system while the estimated tracks are substituted with AR poles in the unknown system. It transforms the metric between these two tracks into the pole-based distance between two dynamic systems, which is discussed explicitly in Section 2.3.

### 2.3. Optimal Subpattern Assignment Metric between AR Models

In this study, the AR models can model the dynamic responses regarding to a vibration system, and a pole-based OSPA distance is proposed as the damage indicator to detect damage. Based on the relationship between the poles and modal parameters [12], natural frequency and damping ratio can be extracted from poles in *z* domain for the *i*th mode (only considering the flexural vibration mode and ignoring the torsional mode according to the simplified shear building assumption):

(7) $$\omega_{i}=\frac{\left|\log\alpha_{i}\right|}{T}=\frac{\sqrt{{\left(\log\left|\alpha_{i}\right|\right)}^{2}+{\left(\arg\alpha_{i}\right)}^{2}}}{T}$$

(8) $$\xi_{i}=\frac{-\log\left|\alpha_{i}\right|}{\omega_{i}\cdot T}$$

where $\omega_{i}$ represents the natural frequency of the *i*th mode,$\;\xi_{i}$ means the damping ratio of the *i*th mode, T denotes the time interval of the system. In this work, the linear elastic method is adopted to model the structural system in different states. The structure in the undamaged and different damaged states can be modeled as a system in a series of different states. In each state, the system can be regarded as linear elastic. Although changing of the structure from undamaged state to damaged state is a nonlinear process, we only focus on the two linear elastic system states, which are the state before damage and after damage, so in this case linear elastic methods still can be used for structural damage detection. The structural natural frequency and damping ratio will change if the structure is damaged, and the relationship between the modal parameters and poles, indicates that the system poles will also change. The pole-based distance reflects the variation between the unknown system and the undamaged system so that it can be served as the structural damage indicator. Firstly, the base distance satisfying three axioms (identity, symmetry, and the triangle inequality), is used to construct the proposed distance measure. Besides, as the damage detection process is a reverse recognition problem without a known priori, the cut-off parameter *c* is omitted. In addition, the original set of target trajectories often contains different target features, such as position, velocity, amplitude, size and shape. If the correlation between different target features is considered, then the Mahalanobis distance can be used as the base distance. However, in this study, since we use AR poles of various orders as multi-dimensional random variables, the correlation between poles of each order is not considered. Thus, the base distance d(:,:) is selected as the Euclidean distance instead of the Mahalanobis distance. The Euclidean distance between two complex poles $\alpha_{i}^{\left(u\right)}$ and $\alpha_{i}^{\left(d\right)}$ in the complex plane is given by:

(9) d(αi(u),αi(d))=((Re(αi(u))−Re(αi(d))))2+(Im(αi(u))−Im(αi(d)))2

where Re(·) and Im(·) mean the real and imaginary part of the poles, respectively; superscript ‘*u*’ and superscript ‘*d*’ depict the undamaged system and damaged system, respectively.

Eventually, a pole-based optimal subpattern assignment distance [54] between AR models is defined by:

(10) $$D\left(\alpha^{\left(u\right)},\alpha^{\left(d\right)}\right)={\left(\frac{1}{q}\left(\underset{\pi \epsilon \prod q}{\min}\overset{q}{\underset{i=1}{\sum }}d{\left(\alpha_{i}^{\left(u\right)},\alpha_{\pi \left(i\right)}^{\left(d\right)}\right)}^{p}\right)\right)}^{1/p}$$

where $\alpha^{\left(u\right)}=\left[\alpha_{1}^{\left(u\right)},\ldots ,\alpha_{q}^{\left(u\right)}\right]$ and $\alpha^{\left(d\right)}=\left[\alpha_{1}^{\left(d\right)},\ldots ,\alpha_{q}^{\left(d\right)}\right]$ represent the poles sets of undamaged system and damaged system, respectively; q indicates the AR order; p∈R and p≥1; $d\left(\alpha_{i}^{\left(u\right)},\alpha_{j}^{\left(d\right)}\right)$ means the base distance mentioned above between two complex poles $\alpha_{i}^{\left(u\right)}$ and $\alpha_{j}^{\left(d\right)}$; ‘∏q’ represents all permutations of the set {1,…,q}; $\alpha_{\pi \left(i\right)}^{\left(u\right)}$ depicts the *i*th pole of $\alpha^{\left(d\right)}$ under permutation case ‘π’, which is yielded by reordering the vector. For instance, by randomly reordering all the elements of the pole set $\left[\alpha_{1}^{\left(u\right)},\alpha_{2}^{\left(u\right)},\alpha_{3}^{\left(u\right)},\ldots ,\alpha_{q}^{\left(u\right)}\right]$, it can produce q! results of permutation, in which one case of the permutations is $\left[\alpha_{q}^{\left(u\right)},\alpha_{3}^{\left(u\right)},\alpha_{1}^{\left(u\right)},\ldots ,\alpha_{2}^{\left(u\right)}\right]$, and this case can be denoted with ‘π’. Hence, the optimization problem of computing the OSPA distance is resolved by finding a minimal sum of all base distances (Equation (9)), which can be accomplished effectively by using the Hungarian algorithm.

### 2.4. Hungarian Algorithm for Optimal Subpattern Assignment 

The Hungarian algorithm is a solution method for the assignment problem, proposed by Kuhn [61], and following the theorem first proposed by the Hungarian mathematician D. Konig. According to the theorem, the maximum number of independent zero elements in the matrix equals the minimum number of lines covering all zero elements. In engineering operating analysis, the Hungarian algorithm serves as a classical algorithm to determine the optimal allocation scheme, resulting in the minimal costs or energies. Herein, the Hungarian algorithm is utilized to solve the pole-based OSPA distance for damage identification in linear dynamic systems.

As for specific assignment problem, there are *q* jobs should be finished by *q* workers, a cost matrix $C_{q\times q}\left(c_{i,j}\right)$ (i,j ϵ {1,…,q}) is defined to determine the optimal assignment for minimum cost, and expressed as:

(11) $$C={\left[\begin{array}{cccc} c_{1,1\;} & c_{1,2\;} & \cdots & c_{1,q\;}\\ c_{2,1\;} & c_{2,2\;} & \cdots & c_{2,q\;}\\ \cdots & \cdots & \cdots & \cdots \\ c_{q,1\;} & c_{q,2\;} & \cdots & c_{q,q\;} \end{array}\right]}_{q\times q}$$

where the element $c_{i,j\;}$indicates the cost of the *j*th job accomplished by the *i*th worker. 

In terms of damage detection problem in this study, the *i*th worker is regarded as the pole $\alpha_{i}^{\left(u\right)}$ in the undamaged system while the *j*th job is compared to the pole $\alpha_{j}^{\left(d\right)}$ in the damaged system, so the element $c_{i,j\;}$ can be replaced with the pole distance $d\left(\alpha_{i}^{\left(u\right)},\alpha_{j}^{\left(d\right)}\right)$ and meanwhile the cost matrix *C* can be transformed into the pole distance matrix $D_{q\times q}\left(d_{i,j}\right)$:

(12) $$D={\left[\begin{array}{cccc} d(\alpha_{1}^{\left(u\right)},\alpha_{1}^{\left(d\right)}) & d(\alpha_{1}^{\left(u\right)},\alpha_{2}^{\left(d\right)}) & \cdots & d(\alpha_{1}^{\left(u\right)},\alpha_{q}^{\left(d\right)})\\ d(\alpha_{2}^{\left(u\right)},\alpha_{1}^{\left(d\right)}) & d(\alpha_{2}^{\left(u\right)},\alpha_{2}^{\left(d\right)}) & \cdots & d(\alpha_{2}^{\left(u\right)},\alpha_{q}^{\left(d\right)})\\ \cdots & \cdots & \cdots & \cdots \\ d(\alpha_{q}^{\left(u\right)},\alpha_{1}^{\left(d\right)}) & d(\alpha_{q}^{\left(u\right)},\alpha_{2}^{\left(d\right)}) & \cdots & d(\alpha_{q}^{\left(u\right)},\alpha_{q}^{\left(d\right)}) \end{array}\right]}_{q\times q}$$

where the pole distance $d_{i,j}=d\left(\alpha_{i}^{\left(u\right)},\alpha_{j}^{\left(d\right)}\right)$ is the base distance defined in Equation (9). Generally, for the sake of using the Hungarian algorithm to calculate the pole-based OSPA distance, there are three common definitions referring to the pole distance matrix D and its assignment:

**Definition** **1.** *Assume the matrix*D*to be a pole distance matrix, a subset consisting of q elements of matrix*D*(without two elements laying on the same row or the same column) is one assignment case for it*.

**Definition** **2.** *The optimal assignment case for the pole distance matrix* D *is obtained by minimizing the sum L of q elements in Definition 1.*

**Definition** **3.** *If all the q elements in Definition 1 are zeros, the corresponding assignment case is called ‘zero-element optimal assignment’*.

Besides, the establishment of the Hungarian algorithm relies on the subsequent two mathematical theorems:

**Theorem** **1.** *If the smallest element of the row (column) of the pole distance matrix*D*is subtracted from each element of the row (column), we can obtain a new matrix*$D^{\prime }$*. The optimal assignment case of the new matrix*$D^{\prime }$*is the same as the one of the original matrix*D*. In other words, these subtractions actually do not change the final optimal assignment case*.

**Theorem** **2.** *If the elements of the pole distance matrix* D *can be divided into two data types: zero and non-zero, the maximum number of independent zero elements (without two zero elements laying on the same row or the same column) in the matrix* D *equals the minimum number of lines covering all zero elements, that is ‘q’. The optimal assignment matrix* $A_{q\times q}\left(a_{i,j}\right)$ *for the pole distance matrix* D *is denoted by:*

(13) $$\underset{A}{\arg\;\min}\left\{L\left(A,D\right)=\overset{q}{\underset{i=1}{\sum }}\overset{q}{\underset{j=1}{\sum }}d_{i,j}a_{i,j}:A\in {\left\{0,1\right\}}^{n\times n}\right\}$$

*where* $a_{i,j}$ *is the decision-making element whose value can be only 1 or 0, i.e., if* $a_{i,j}=1$ *, it means adopting the pole distance* $d_{i,j}$ *of the pole distance matrix* D *to calculate the sum L, otherwise if* $a_{i,j}=0$ *, it means the pole distance* $d_{i,j}$ *is not selected for calculation of the sum L. To obtain the optimal assignment for pole-based OSPA distance between AR models of the undamaged system and the damaged system, main steps using the Hungarian algorithm are summarized as follows:*

*Step 1: Construct the pole distance matrix* D *according to Equation (12);*

*Step 2: According to Theorem 1, subtract the smallest element from each row of matrix*D*, and obtain the new matrix*$D^{\prime }$;

*Step 3: According to Theorem 1, subtract the column minimum element from each column of matrix*$D^{\prime }$*, and obtain the new matrix*$D^{\prime \prime }$;

*Step 4: Use the appropriate row or column with the minimum number to cover all zeros in the new matrix* $D^{\prime \prime }$ *, and the minimum number will be used for subsequent steps;*

*Step 5: Judge whether it is an optimal assignment according to Theorem 2. If the minimum number of these covering lines is ‘q’, a zero-element optimal assignment in Definition 3 can be obtained. Substitute all the ‘0’ elements (without two elements laying on the same row or the same column) with ‘1’, the rest elements of the new cost matrix* $D^{\prime \prime }$ *are changed to be ‘0’, and the optimal assignment matrix* A *is acquired. Else, if the minimum number of the covering lines is less than q, it cannot find a zero-element optimal assignment, continue to the next step;*

*Step 6*: Find the minimum element from the uncovered elements of matrix $D^{\prime \prime }$*, subtract the minimum element from all uncovered elements, add the minimum element to the element covered by both horizontal and vertical lines, and then go back to Step 4*.

**Figure 1:**
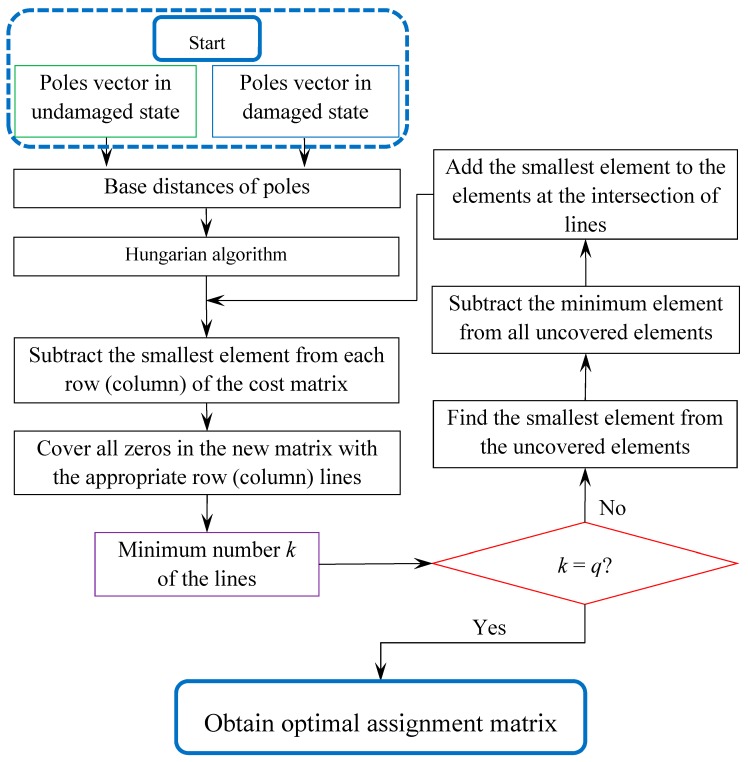
Flowchart of Hungarian algorithm for optimal assignment.

The flowchart of the Hungarian algorithm can be described in Figure 1. Finally, the optimal assignment matrix, A, is provided by the Hungarian algorithm. The OSPA distance between AR models of two sets of poles representing the undamaged and damage systems is obtained by Equation (10).

## 3. Procedure for Damage Localization and Quantification

In most cases, the excitations in civil engineering, such as wind and earthquakes, are mutually dependent and correlated while acting on structures. A de-correlation procedure [34] is adopted to remove spatial correlation of the excitations on different sites. Firstly, the *m* dimensional sensor signals x(t) with a zero mean are obtained by removing the linear trend from the original signals. Then, they are pre-processed by using the following whitening transformation:

(14) y(t)=Wx(t)

where y(t) denotes the signals whitened, and W indicates a matrix of m×m for whitening, which is to change the covariance matrix $E\left\{y\left(t\right)y{\left(t\right)}^{T}\right\}$ to be the unit matrix $I_{m}$; the required decorrelation matrix can be calculated as:

(15) $$W=\varphi_{x}{}^{-1/2}\Theta_{x}^{T}=diag\left\{\frac{1}{\sqrt{\lambda_{1}}},\frac{1}{\sqrt{\lambda_{2}}},\ldots ,\frac{1}{\sqrt{\lambda_{m}}}\right\}\Theta_{x}^{T}$$

where $\Theta_{x}$ indicates an orthogonal matrix and $\varphi_{x}=diag\left\{\lambda_{1},\lambda_{2},\ldots ,\lambda_{m}\right\}$ means a diagonal matrix with positive eigenvalues $\lambda_{1}\geq \lambda_{2}\geq \ldots \geq \lambda_{m}>0$.

In order to take the operational and environmental uncertainty into account, an undamaged limit (UL) is introduced in this study. The time history of response in the undamaged state can be divided into *n* subsequences, which are modeled with *n* AR models, representing by *n* AR poles $\left[\alpha_{1}^{\left(u\right)},\alpha_{2}^{\left(u\right)},\ldots ,\alpha_{i}^{\left(u\right)},\ldots ,\alpha_{j}^{\left(u\right)},\ldots ,\alpha_{n}^{\left(u\right)}\right]$. The proposed UL is defined as follows:

(16) $$\mathrm{UL}=\frac{\sum_{j=1}^{n}\sum_{i=1}^{n}d\left(\alpha_{i}^{\left(u\right)},\alpha_{j}^{\left(u\right)}\right)}{n^{2}}$$

where the number *n* is determined by the experience of engineers, $d\left(\alpha_{i}^{\left(u\right)},\alpha_{j}^{\left(u\right)}\right)$ is the Euclidean distance between $\alpha_{i}^{\left(u\right)}$ and $\alpha_{j}^{\left(u\right)}$, and the authors would suggest it to be more than 8 normally to cover the uncertainty of measurements. Thus, the UL is determined by the mean value of the pole-based OSPA distances between multiple AR models of undamaged systems. 

**Figure 2:**
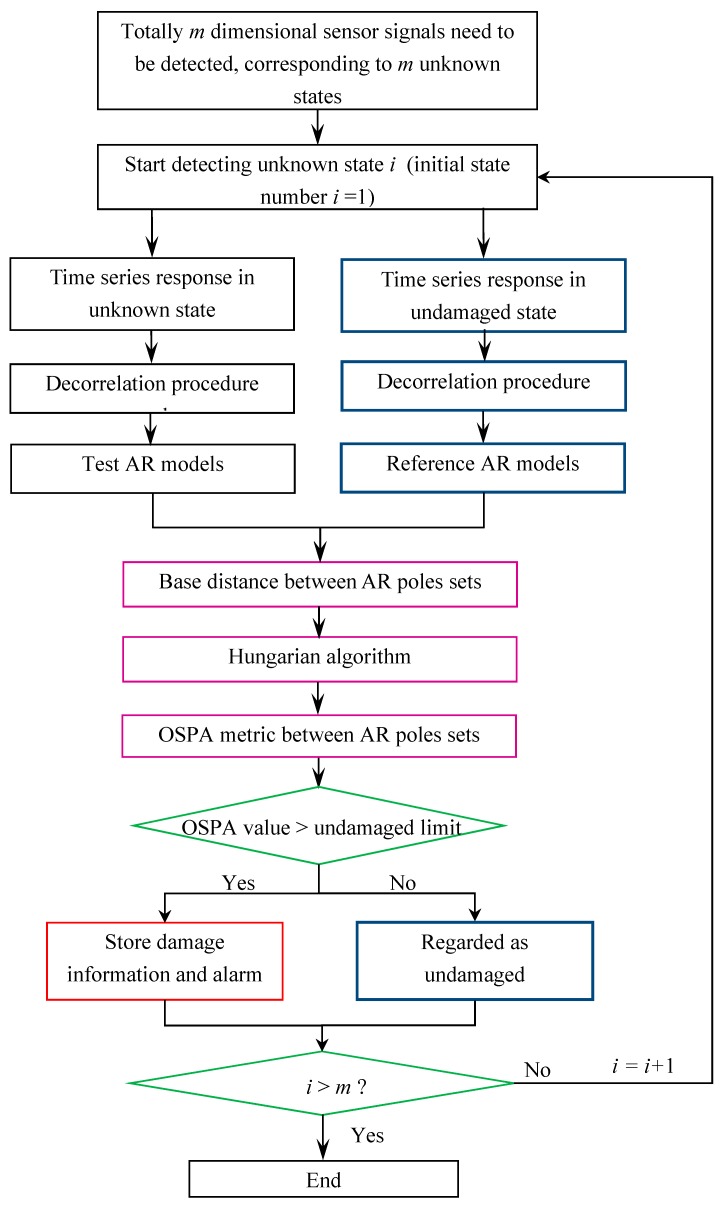
Flowchart of the proposed damage detection methodology.

The steps of the proposed damage detection method can be summarized in a flowchart shown in Figure 2. Firstly, there are totally *m* dimensional sensor signals from the structure to be evaluated, corresponding to *m* unknown states. State number *i* represents the *i*th dimension of the sensor signal, while the whole detection process starts from *i* = 1 and end at *i* = *m*. Time series responses such as accelerations from the undamaged structure and structures in unknown states are preprocessed with a decorrelation procedure and then used to construct the AR models. Secondly, the Burg’s method is adopted to get estimated parameters from the AR models, and then the poles of reference and test systems are solved. Thirdly, the Hungarian algorithm is applied to derive the pole-based OSPA distance. If the identified OSPA distance is larger than the UL, the system is judged as ‘damaged’, and the severity of damage is quantified by the magnitude of OSPA. Otherwise, if the identified OSPA distance is smaller than the UL, the system can be regarded as ‘undamaged’. Finally, damage identification of different damage cases can be achieved according to the magnitude of damage indicator, which includes the information of location and quantification of damages. In addition, in order to show the advantage of the proposed damage indicator based on OSPA distance compared with the classical damage indicator, meanwhile, the damage detection using the change of natural frequencies is conducted for the following case studies. The change of natural frequencies is defined as:

(17) $$\Delta f_{i}=f_{i}^{u}-f_{i}^{d}$$

where$\;f_{i}^{u}$ indicates the *i*th natural frequency of the undamaged structure,$\;f_{i}^{d}$ depicts the *i*th natural frequency of the damaged structure, $\Delta f_{i}$ represents the change of the *i*th natural frequency of the structure.

## 4. Case Study I: Five-Story Shear Building Model

### 4.1. Numerical Analysis

**Figure 3:**
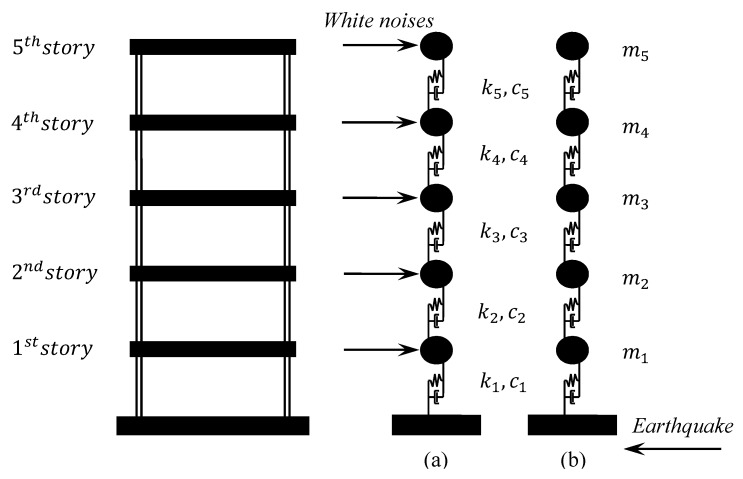
Five-story shear building model subjected to: (**a**) mutually correlated inputs of white noises; (**b**) earthquake excitation.

As depicted in Figure 3, a five-story sheared building model, which can be simplified as a 5-DOF system, is used to verify the performance of the proposed damage detection method, whose results are compared with the change of natural frequencies. In the first case, the structural system is subjected to mutually correlated exogenous inputs of white noises on every mass. Besides, in order to consider the feasibility and performance of the proposed damage indicator applied to the structure with nonstationary excitation, the structural system is excited by the El Centro earthquake acceleration record in another simulation case. 

**Figure 4:**
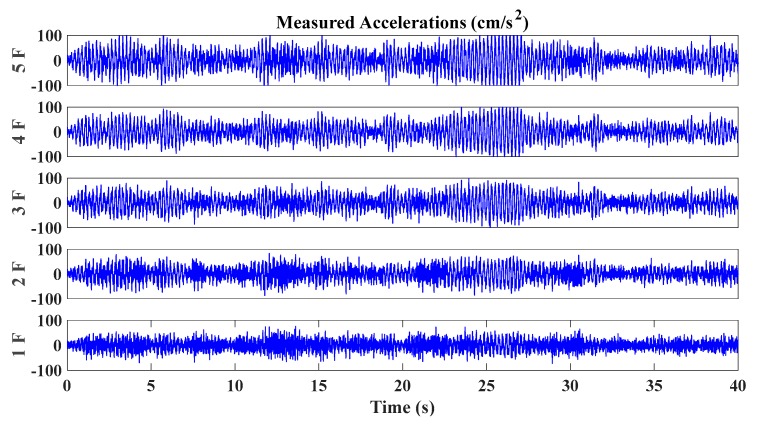
Time histories of simulated acceleration excited by mutually correlated white noises.

**Figure 5:**
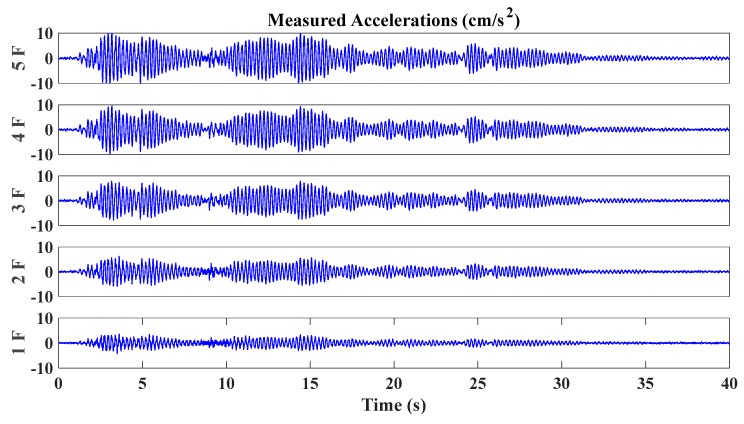
Time histories of simulated acceleration excited by El Centro earthquake record.

Relevant structural parameters are given as follows: the mass of every story is 1 × 10^4^ kg, and the lateral stiffness is 1 × 10^8^ N/m; the damping ratio is assumed to be 1% for all modes; the first five natural frequencies of the simulated shear model are given as 4.53 Hz, 13.22 Hz, 20.84 Hz, 26.78 Hz and 30.54 Hz; the data sampling frequency was 100 Hz, and the time duration of each story response is 40 s. Considering the disturbance of environmental influence, 5% noise level is added into the acceleration data. In this study, we only consider the damage occurring on one single floor, and the structural damage is simulated by the inter-floor stiffness reduction. Each damage case is a single floor damage with one certain damage degree. Totally we design five different damage locations corresponding to five floors respectively, with five degrees of damage (10%, 20%, 30%, 40% and 50% reduction of inter-floor stiffness) occurring on each floor. Thus, there are totally 5 × 5 = 25 damage scenarios. The simulated stiffness degradation is a typical damage mode for buildings, which can represent a general situation in the SHM of civil structures [64,65,66]. Figure 4 and Figure 5 illustrate the typical acceleration time histories of all stories of the simulated shear building model under mutually correlated white noises and El Centro earthquake excitation, respectively.

The first five natural frequencies of this structure in undamaged state calculated by the stochastic subspace identification method are given as 4.53, 13.22, 20.85, 26.78 and 30.54 Hz. Since the reduction of structural stiffness leading to decreasing of natural frequencies, the change of natural frequencies between undamaged structures and damaged structures can serve as a damage indicator.

**Figure 6:**
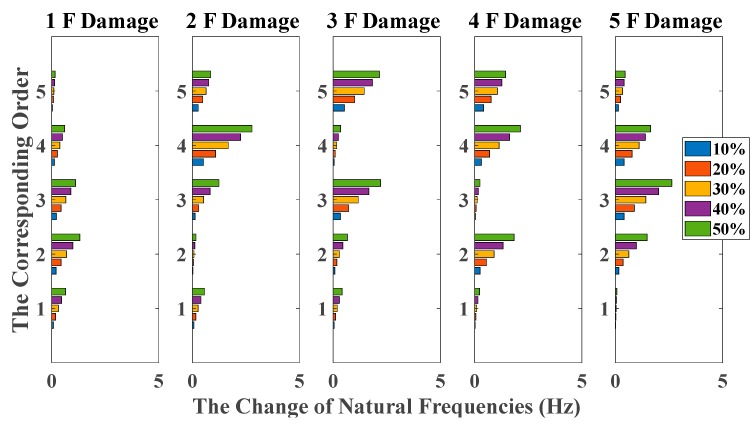
Damage detection results based on the change of natural frequencies (mutually correlated white noises inputs, 5% noise, data length = 4000).

**Figure 7:**
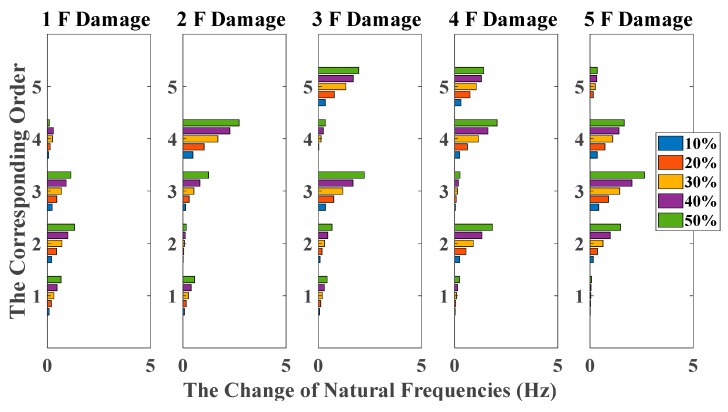
Damage detection results based on the change of natural frequencies (El Centro earthquake excitation, 5% noise, data length = 4000).

Figure 6 and Figure 7 give the amount of natural frequencies declining in all damage scenarios, corresponding to the case of white noises excitation and El Centro earthquake excitation, respectively. However, due to natural frequencies only representing the global structural property, it cannot provide a distinct relationship between damage locations and the corresponding order of natural frequencies. Therefore, it cannot achieve damage localization in this case.

Additionally, the UL of each story is independently calculated through the mean value of OSPA distances between ten data sets in undamaged states, and the time duration of each data set is 40 s. Given the scenarios with damage occurring on the first floor with different damage degrees as an example, pole-based OSPA distances and their corresponding parameters are listed in Table 1 and Table 2, corresponding to the case of white noises excitation and El Centro earthquake excitation, respectively. 

**Figure 8:**
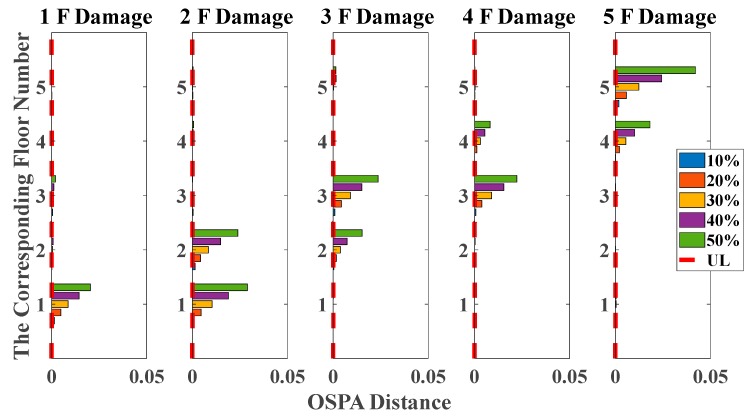
Damage detection results based on the OSPA distance (mutually correlated white noises inputs, 5% noise, autoregressive model, data length = 4000, AR order = 1, OSPA.p = 2).

**Figure 9:**
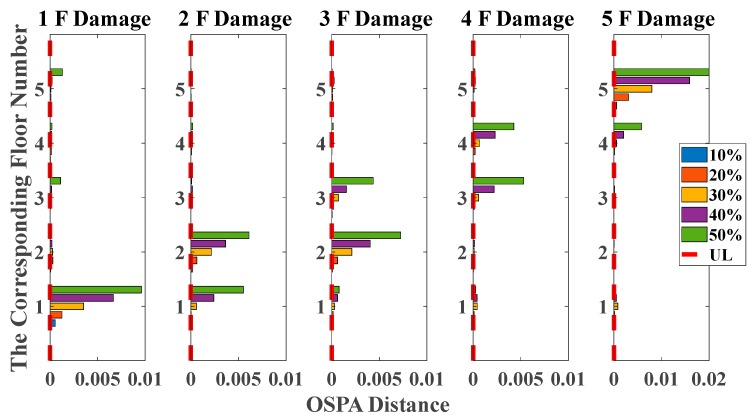
Damage detection results based on the OSPA distance (El Centro earthquake excitation, 5% noise, autoregressive model, data length = 4000, AR order = 1, OSPA.p = 2).

As shown in Table 1 and Table 2, the OSPA distances are observably higher on the first floor than others. Moreover, damage identification results based on the OSPA distances of all damage scenarios are shown in Figure 8 and Figure 9, reflecting evident regularity in the damage location. For instance, when the stiffness of the third story is reduced, the responses of the second and third floors are more severely affected than other floors, so that the OSPA values of the second and third floors are much larger than the other floors. Moreover, it can be obviously seen that the damage indicator can clearly quantify the damage, proportionally increasing with the damage extent. It should be noticed that the proposed damage indicator can obtain satisfactory damage identification results even under non-stationary excitation conditions, but the results are still not as stable enough as those of white noises excitation cases, especially in the case of 10% and 20% damages. This phenomenon is probably due to the non-stationary property of the time series, which brings some unavoidable limitations for AR coefficients estimation using Burg’s method, so it still needs further study to solve this problem in the future. 

**Figure 10:**
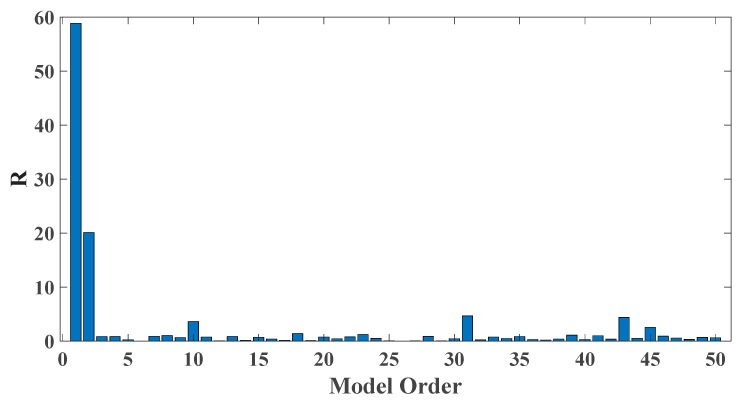
Evaluation index of damage detection results using various AR orders under the case of 30% damage occurring on the third floor (mutually correlated white noises inputs, 5% noise).

**Figure 11:**
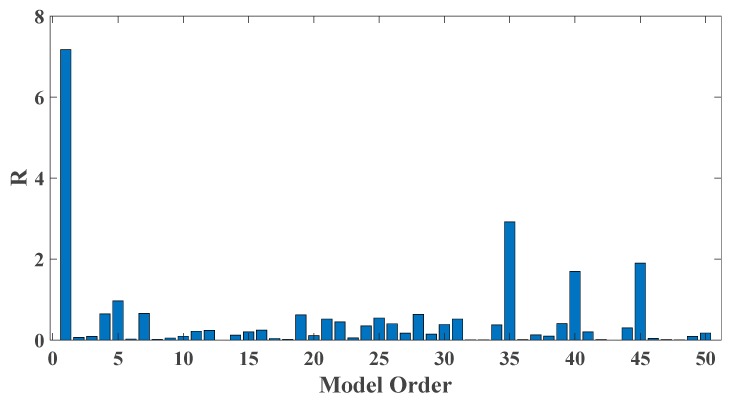
Evaluation index of damage detection results using various AR orders under the case of 30% damage occurring on the third floor (El Centro earthquake excitation, 5% noise).

**Figure 12:**
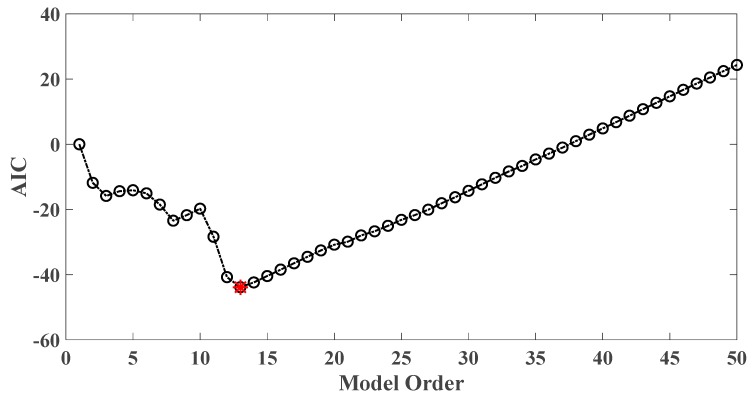
AIC of an AR model for the third floor response under the case of 30% damage occurring on the third floor (mutually correlated white noises inputs, 5% noise).

**Figure 13:**
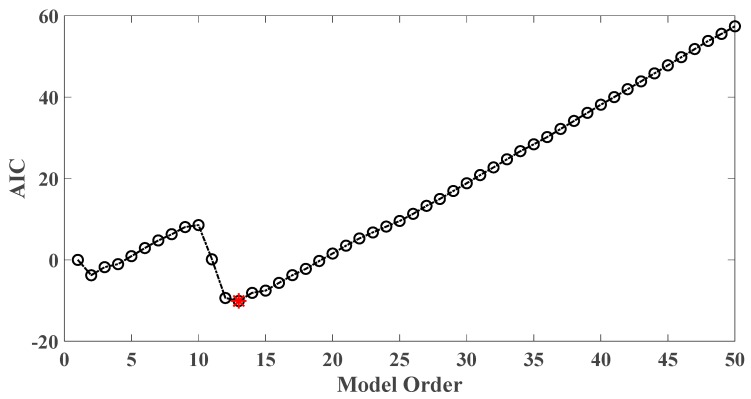
AIC of an AR model for the third floor response under the case of 30% damage occurring on the third floor (El Centro earthquake excitation, 5% noise).

In order to assess the influences on damage detection results using AR models with different orders, the following investigation is conducted. Firstly, given the scenario of 30% damage occurring on the third floor as an example, an index *R* for evaluating damage detection results is defined as follows:

(18) $$R=\frac{d_{2}+d_{3}}{d_{1}+d_{4}+d_{5}}$$

where $d_{i}$ denotes the OSPA value of the *i*th floor (1≤i≤5). If the result of damage localization is correct, the value of the numerator in Equation (18) would be much larger than the denominator, so the value of R would be rather large meanwhile. Otherwise, if the value of the numerator in Equation (18) is much less than the denominator, then the value of *R* would be very small, indicating an unsatisfactory identification result. Therefore, the optimal order for ideal damage detection is corresponding to the highest *R* value. With changing the AR order from 1 to 50, all the corresponding *R* values are shown in Figure 10 and Figure 11. Normally, the selection of AR order obeys the AIC criterion. Given the time series response of the third floor (the case of 30% damage occurring on the third floor) as an example, as shown in Figure 12 and Figure 13, the AIC criterion indicates that the optimal model order is 13 for both cases of white noises excitation and El Centro earthquake excitation. However, it can be clearly observed that the most considerable *R* value is obtained by selecting AR order = 1, while choosing 13 as the AR order (according to AIC criterion) cannot give an appropriate identification result. Due to the limitation of AIC criterion in this case, we suggest selecting AR order = 1 instead of 13 as the optimal model order.

**Figure 14:**
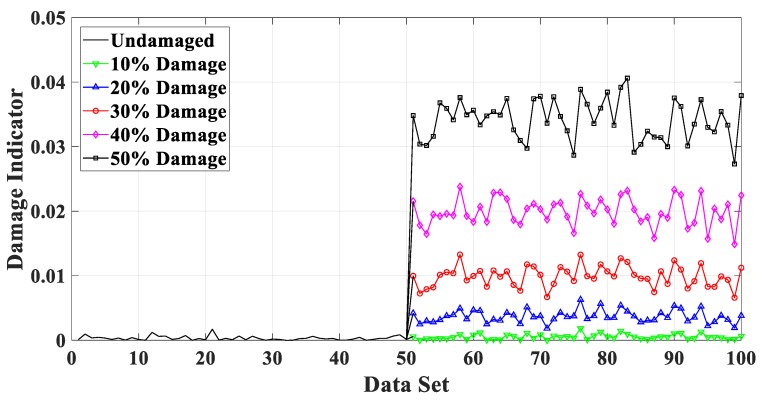
Damage detection for sudden structural change.

In a practical health monitoring system, the time series response of all floors are generally recorded at regular intervals, with two minutes, one hour or one day. In order to investigate the feasibility of the proposed damage indicator embedded in the structural health monitoring system to detect system changes, numerical simulations of continuously detecting damage of the abovementioned shear building structure at the same time intervals are conducted. Three hundred data sets are simulated and each comprises 4000 sampled data (at a sampling period of 0.01 s, hence a total of 40 s for each data set). The former 50 data sets are simulated acceleration time histories in undamaged state, while the latter 250 ones are in damaged states with different damage extent (10% through 50%). Given the scenario of the fifth story damage as an example, the identification results are shown in Figure 14. During the first 50 data sets, since the structural state does not change, the damage indicator is close to zero. As for the later 250 data sets, it shows a clear damage quantification with the growing damage degrees. As a result of the estimated deviation of AR poles for different data sets, it shows a continuous undulation during the undamaged process and the damaged process. Still, overall the damage indicators fluctuate at a certain mean value and the whole trend indicating the sudden system change is distinct. Thus, the proposed damage indicator is promising to contribute to the design of SHM systems.

### 4.2. Experimental Validation

**Figure 15:**
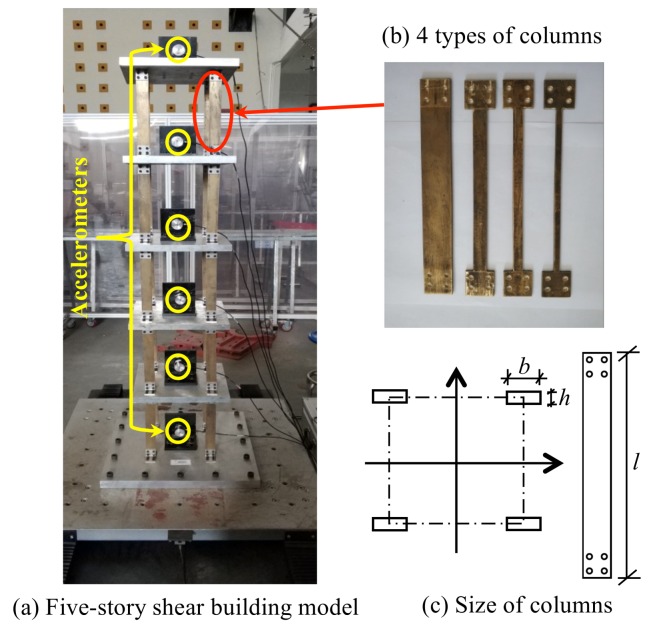
Experimental setup of five-story shear framework model. (**a**) Five-story shear building model (**b**) 4 types of columns. (**c**) Size of columns.

**Figure 16:**
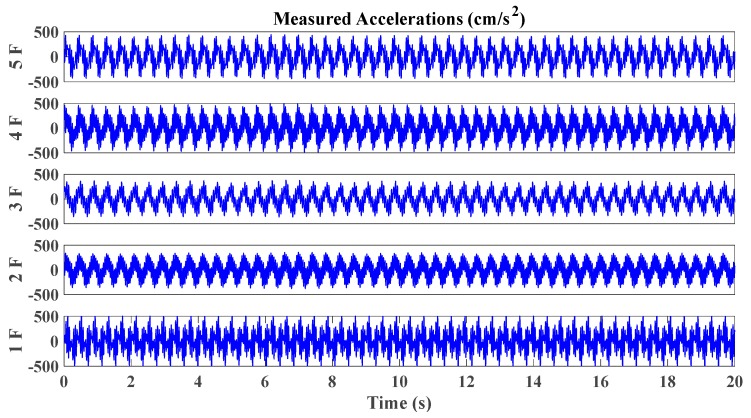
Typical acceleration time histories of small-scale shear framework structure excited by sine sweeping-frequency excitation.

A small-scale shear frame structure of five stories is presented in Figure 15a, placing on a shake table. The story mass is made of aluminum floor slabs, bronze columns (Figure 15b) and sensors. Young’s modulus of bronze is 1.0 × 10^11^ N/m^2^. The mass of the 1st–4th story is 7.2523 kg, while the 5th story is 6.5421 kg. The inter-floor stiffness is controlled by the bronze plate springs, shown in Figure 15c. Relevant size parameters of each column are shown in Table 3. The healthy state corresponds to initial columns intact (column type 0). In this study, we only consider the scenario with the damage occurring on one single floor, which is imitated by replacing four original columns on one certain floor with the weaker ones of three different types. Thus, there are totally 5 × 3 = 15 damage scenarios investigated in this experiment. Three types of weaker columns used in the experiment are shown in Figure 15b and Table 3, corresponding to the cases of comparatively damage level 1 (53.33%), damage level 2 (66.67%) and damage level 3 (80.00%). The force excitations to the structure are provided with a unidirectional electric shaking table. The data acquisition equipment used in this study is a Donghua DH8303 dynamic signal acquisition system. Six sensors used for the measurement of excitation and structural responses are the 1A202E IEPE piezoelectric accelerometers, whose sensitivities are 996.1, 987.7, 992.3, 1003, 1001 and 992.5 mV/g, respectively. The maximum acceleration measurement range is 5 g. Accelerometers are installed on the center of each floor plate to measure the acceleration response, while one is installed on the basement to measure the input. A sinusoidal wave is used as the input signal, with frequencies sweeping from 1.0 to 15.0 Hz with a growth rate of 0.5 Hz/min. The sampling frequency of acceleration time histories is 200 Hz. A part of structural acceleration time history measured in the undamaged state is shown in Figure 16.

**Figure 17:**
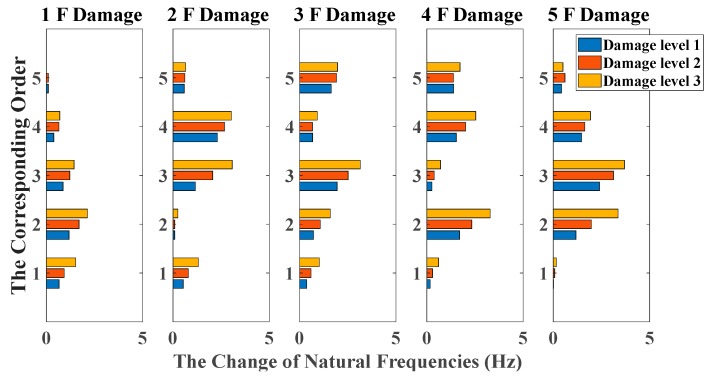
Damage detection results based on the change of natural frequencies (sine sweeping-frequency excitation, data length = 4000).

**Figure 18:**
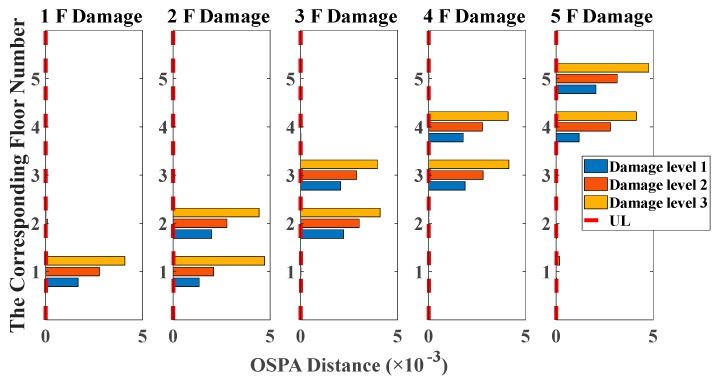
Damage detection results based on the OSPA distance (sine sweeping-frequency excitation, autoregressive model, data length = 4000, AR order = 1, OSPA.p = 2, Assignment = 1).

By using the stochastic subspace identification method, the first five natural frequencies of this experimental structure in the undamaged state are identified and obtained as 3.54, 10.67, 17.15, 22.00 and 25.25 Hz. Figure 17 shows the decreasing of the first five natural frequencies in all damage cases. Nevertheless, it fails to reflect a clear damage localization pattern since there is no distinct relation between damage locations and the corresponding order of natural frequencies. On the other hand, the UL of each story is independently calculated through the mean value of OSPA distances between ten data sets in undamaged states, and the time duration of each data set is 20 s. The pole-based OSPA distance, and its relevant parameters for the case of first-floor damage, are shown in Table 4 as an example. As a result, the OSPA distances are distinctly higher on the damaged location than the undamaged location. In Figure 18, the OSPA distances visibly indicate the location of all damages. For instance, after the lateral stiffness of the columns connecting the second and third floors is reduced, the floor responses of the second and third floors are more severely affected than the other floors, which leads to that OSPA values of the second and third floors are much larger than the other floors. Therefore, the proposed damage indicator takes more advantages of damage localization than the classical indicator of change in natural frequencies. In addition, the damage indicator concomitantly grows with the damage extent increasing, showing an effective quantification ability of structural damages. 

**Figure 19:**
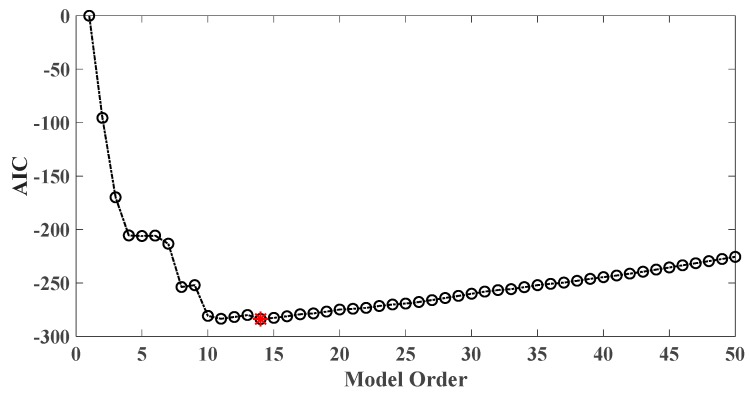
AIC of an AR model for the third floor response under the case of level 2 damage occurring on the third floor.

**Figure 20:**
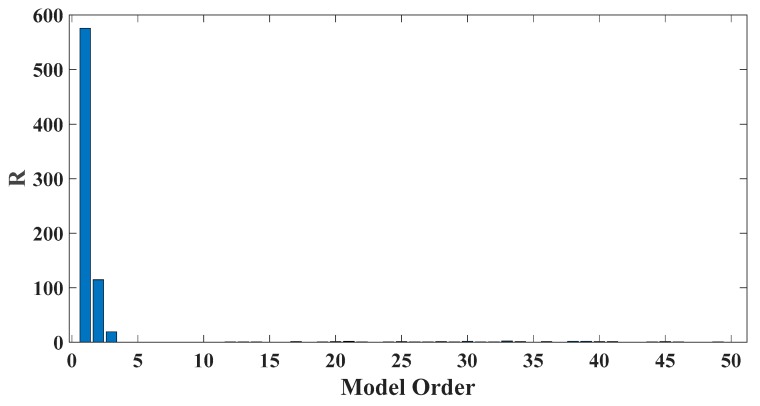
Evaluation index of damage detection results using various AR orders (the case of level 2 damage occurring on the third floor).

Taking damage level 2 existing on the third floor as an illustrative example, the AIC plot of the third floor response modeled with AR model is shown in Figure 19, while R values of various AR orders are given in Figure 20.

As a result, AR order = 1 is the optimal model order for the best damage detection results. In contrast, AR order = 14 is believed to be optimal according to AIC criterion while it does not yield satisfactory damage identification results.

## 5. Case study II: Large-Scale Shear Frame Structure

### 5.1. Model Description

**Figure 21:**
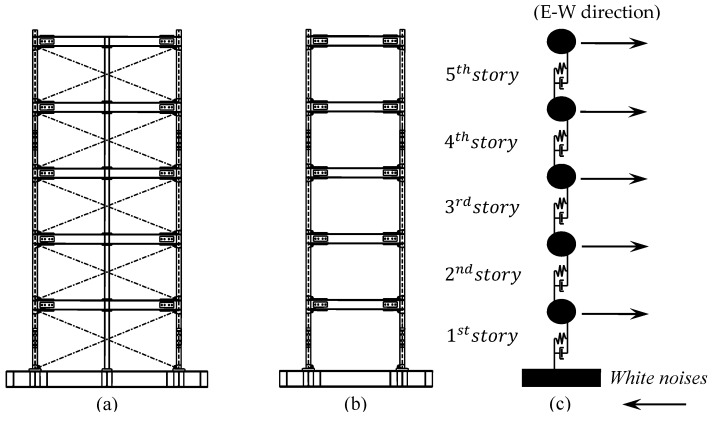
Experimental building model: (**a**) E-W direction; (**b**) N-S direction; (**c**) Simplified shear building model.

In this section, the proposed method is evaluated by a large-scale shear frame construction, which was conducted by the Building Research Institute of Japan [67]. The experimental building schematic is depicted in Figure 21. The mass of every story is 2.57 ton and story heights are all 1 m. The length of the long side is 3 m, while that of the short side is 2 m. The natural frequencies and damping ratios of the original structure were identified using the ARX model [68], as shown in Table 5. The shake table excites the structure using a white noise with bandwidth [0, 200] Hz along the long-side direction. One Accelerometer is installed on every story to record the acceleration response along the long-side direction. The acceleration time histories are recorded for 40.92 s with the sampling period 0.005 s, which obtains each record of 8192 sampled data. Similar to the simulation cases mentioned above, it can be simplified as a five-DOF shear model. It was loaded by white noises on every floor, as shown in Figure 21c. 

**Figure 22:**
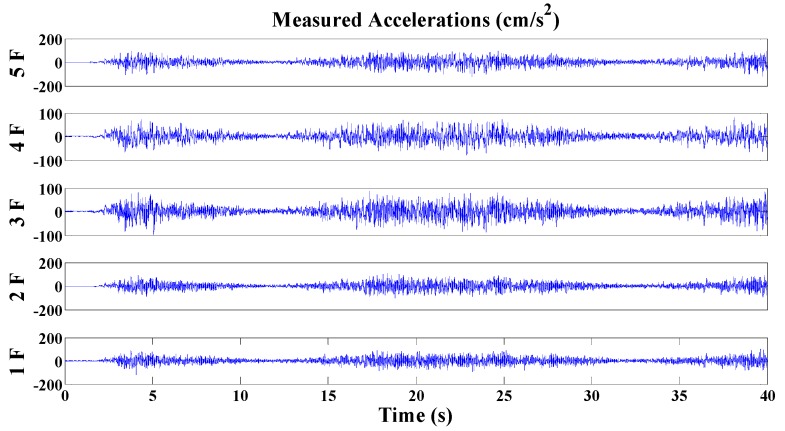
Acceleration time histories of the large-scale framework model excited by a shaking table.

The corresponding structural response of each story is shown in Figure 22. In this experiment, structural damages are simulated by removing the braces or central columns on a single floor (the first, third or fifth floor). 

**Figure 23:**
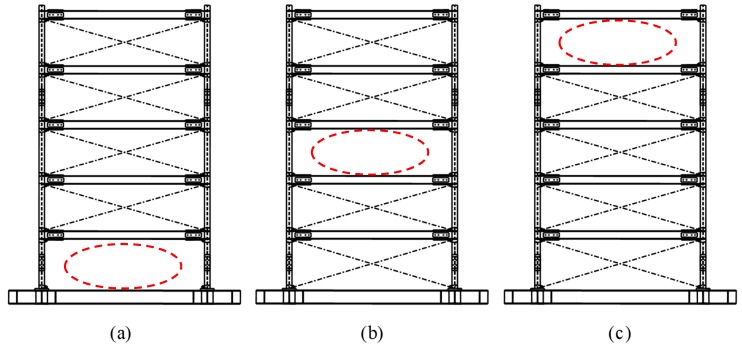
Damage cases: removing braces on the (**a**) first; (**b**) third; and (**c**) fifth story.

**Figure 24:**
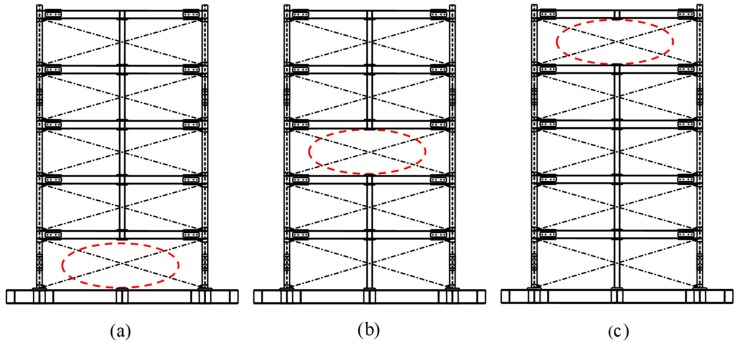
Damage cases: removing central column on the (**a**) first; (**b**) third; and (**c**) fifth story.

For the damage case ‘removing braces’, the corresponding undamaged structure is the test frame structure without central columns (as is shown in Figure 23). But for the damage case ‘removing columns’, the corresponding undamaged structure is the test frame structure with central columns (as shown in Figure 24). 

### 5.2. Analysis of Results

**Figure 25:**
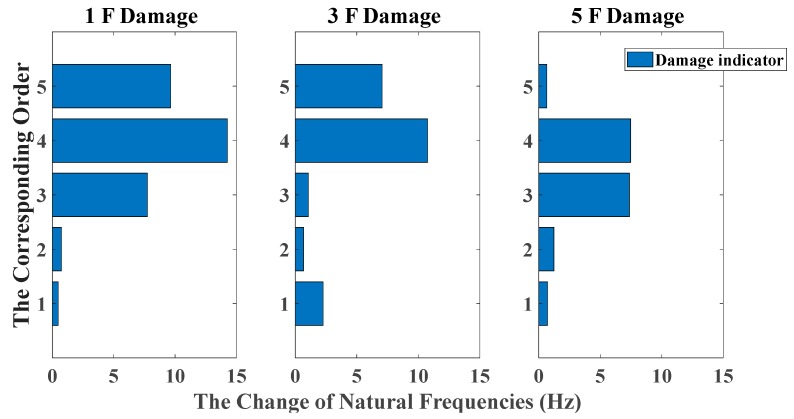
Damage detection results based on the change of natural frequencies (removing braces, white noises excitation, data length = 4000).

**Figure 26:**
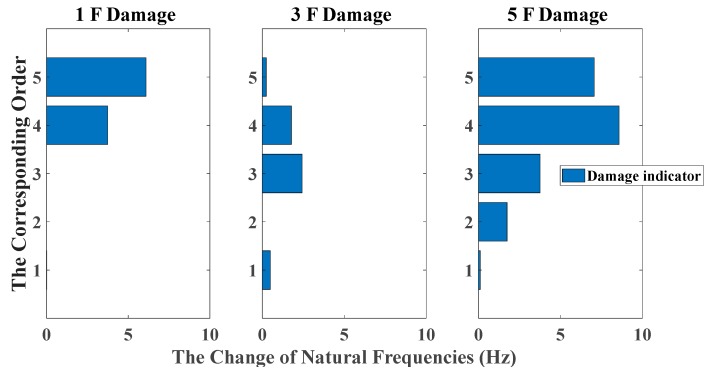
Damage detection results based on the change of natural frequencies (removing central columns, white noises excitation, data length = 4000).

Figure 25 and Figure 26 show the degradation of the first five natural frequencies in all damage cases, whereas it is unable to locate the actual damage because of its unclear relationship between damage locations and the corresponding order of natural frequencies. That is the reason why we need to introduce the pole-based damage indicator for accurate identifications. The UL of each story is independently calculated through the mean value of OSPA distances between eight data sets in undamaged states. The time duration of each data set is 5 s. 

**Figure 27:**
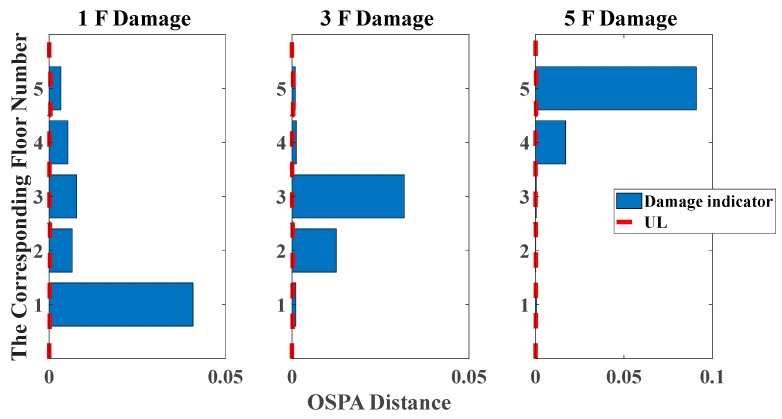
Damage detection results based on the OSPA distance (removing braces, white noises excitation, autoregressive model, data length = 4000, AR order = 2, OSPA.p = 2).

**Figure 28:**
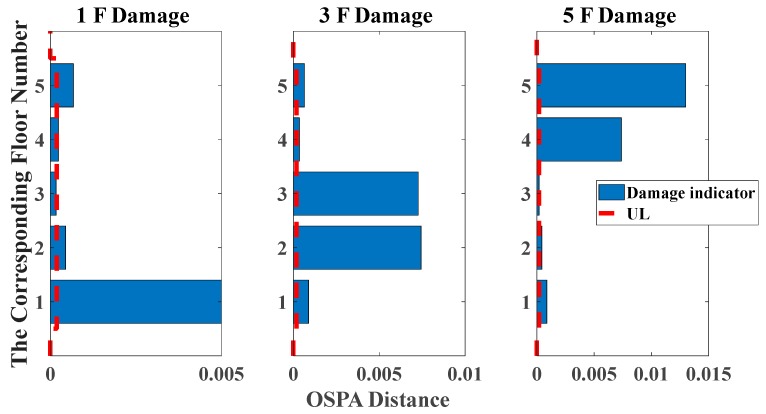
Damage detection results based on the OSPA distance (removing central columns, white noises excitation, autoregressive model, data length = 4000, AR order = 2, OSPA.p = 2).

Analogously, the pole-based OSPA distance and its relevant parameters using the Hungarian algorithm for the case of removing braces and columns are shown in Table 6 and Table 7. From the assignment of Table 6 and Table 7, a rational selection among the elements of the pole distance matrix is used for OSPA values calculation, avoiding the spurious damage quantification. For the sake of showing the superiority of damage localization compared with the change in natural frequencies, as shown in Figure 27 and Figure 28, the OSPA distances are calculated for three damage scenarios. The results show that considerable OSPA distances exist at the locations where the columns or braces have been removed. For instance, after removing the braces or columns linking with the second and third floors, the floor responses of the second and third floors are more severely affected compared with the other floors, such that the larger OSPA values occur on the second and third floors than on the other floors. The results show that the proposed pole-based OSPA distance is capable of identifying the damage locations and extents, which provides the possibility of further practical application in SHM system.

**Figure 29:**
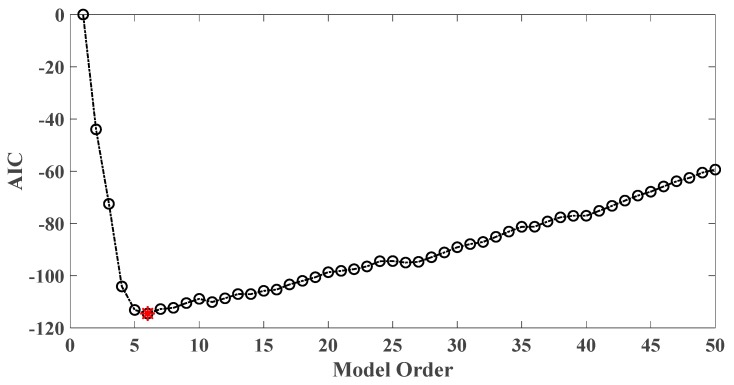
AIC of an AR model for the third floor response under the case of removing braces on the third floor.

**Figure 30:**
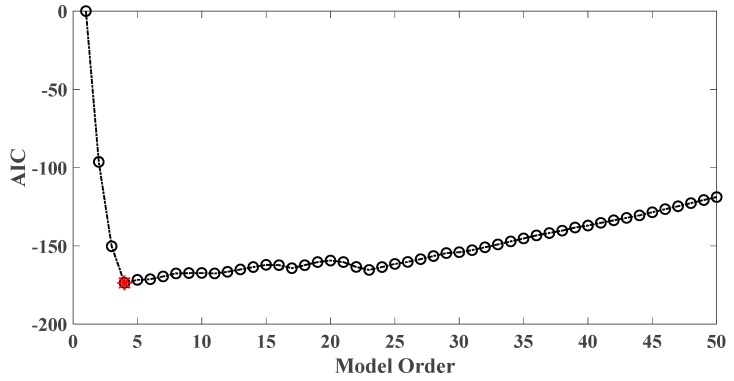
AIC of an AR model for the third floor response under the case of removing central columns on the third floor.

**Figure 31:**
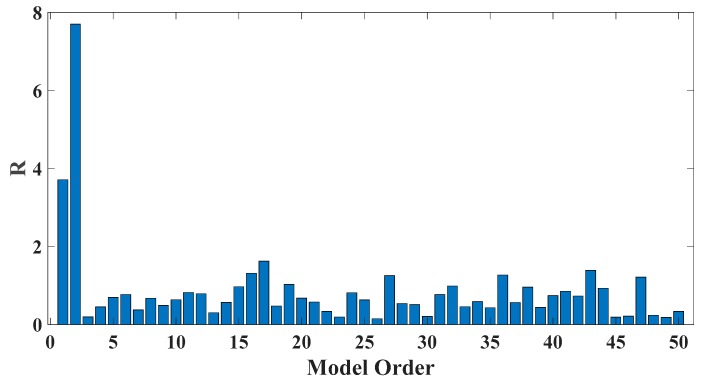
Evaluation index of damage detection results using various AR orders (the case of removing braces on the third floor).

**Figure 32:**
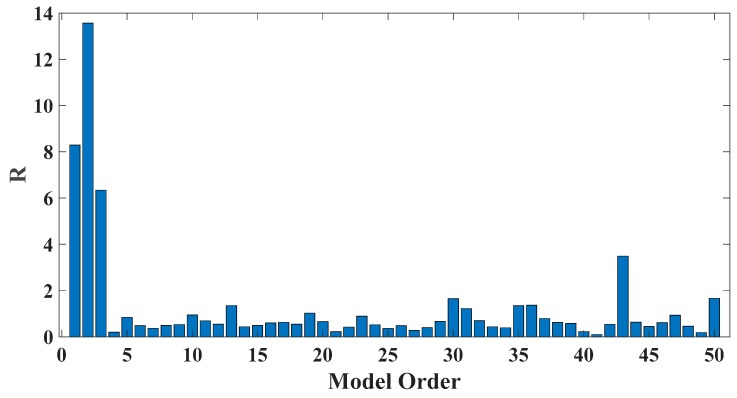
Evaluation index of damage detection results using various AR orders (the case of removing central columns on the third floor).

Two examples illustrating the impact of changing AR order on damage detection results are given in the case of removing braces and central columns on the third floor. Figure 29 and Figure 30 show the AIC plot of the third-floor response modeled with AR model. At the same time R values of various AR orders from 1 through 50 are given in Figure 31 and Figure 32. Finally, AR order = 4 or 6 is believed to be optimal based on the AIC criterion but does not yield satisfactory identification results. In comparison, it is more reasonable to choose AR order = 2 as the model order for best damage identification. It should be pointed out that selecting the appropriate model order is crucial for the success of damage detection. Still, due to the diversity of responses and the complexity of the damage scenarios, it is difficult to give a general guideline for choosing proper AR order. Since the R-value criterion is only for single floor damage scenario of the shear building structure, it is still necessary to establish a general criterion for model order selection in the future research.

## 6. Conclusions

To improve the insufficient damage localization ability and computing flexibility of previous methods, this study established a novel output-only structural damage indicator defined as the pole-based OSPA distance between AR models to identify damages of shear structures. The damage identification process introduces the concept of multi-target tracking, which uses the Hungarian algorithm to calculate the pole-based OSPA distance. Numerical analysis and experimental tests using a five-story shear building model have been conducted to test the feasibility and effectiveness of the improved damage indicator. The results demonstrate the robust stability of the proposed method through effective detection and quantification of structural damages.

Different from the modal parameter-based damage indicators only reflecting the change of the overall structural characteristics, the proposed pole-based OSPA distance aims at local damage identification in a data-driven way. The proposed damage indicator only needs the information of structural responses without requiring a priori knowledge about the inputs of the structure, which effectively addresses the challenge of unknown excitation in practical applications. Also, compared with the pattern recognition based damage indicator relying on tremendous training data, complicated pattern recognition tools and complex physical models, the proposed damage indicator is more flexible in the calculation process. By tracking the change of the poles extracted from structural responses, it can provide another practical idea for damage identification, which indicates the promising applications of embedding the proposed damage indicator in the SHM system. It is worth noting that the proposed structural damage indicator is an innovative application of multi-target tracking in structural damage identification, which reflects the interdisciplinary integration, and promotes the prospective application of more target tracking algorithms in the field of damage detection in complex engineering structures. 

In addition, most of the structures in engineering fields are actually composed of heterogeneous materials. Real structural damages such as concrete cracking and steel corrosion often have nonlinearities that are more complex. Hence, there has been a perceived need for further investigation about the use of the proposed damage indicator to identify the nonlinear damages in complex engineering structures. Besides, in order to tackle the identification problem of continuously degrading process which is crucial for real-time online SHM system, it is necessary to develop a real-time damage indicator by combining the on-line monitoring technique (such as unscented Kalman filter) with the proposed pole-based metric in the future research. Moreover, since this work only use the acceleration responses to extract system poles as damage sensitive features, it is promising that the pole-based OSPA distance can be enhanced with the combination of various kinds of structural responses including acceleration, displacement, strain, etc. In this way, it is possible to achieve more comprehensive and accurate structural damage assessment for complex structures in real engineering fields.

## Figures and Tables

**Table 1:** OSPA distance and the corresponding parameters for the case of damage occurring on the first floor (mutually correlated white noises inputs, 5% noise, autoregressive model, data length = 4000, AR order = 1, OSPA.p = 2, Assignment = 1).

Damage (%)	Floor	Pole	D	OSPA Distance
10	1	0.0845	1.4323 × 10^−3^	0.0014
0	2	0.2531	1.7889 × 10^−4^	0.0002
0	3	0.2286	5.3499 × 10^−4^	0.0005
0	4	0.2405	7.6139 × 10^−5^	0.0001
0	5	0.5691	1.3142 × 10^−4^	0.0001
20	1	0.1167	4.9122 × 10^−3^	0.0049
0	2	0.2563	2.7335 × 10^−4^	0.0003
0	3	0.2219	2.6851 × 10^−4^	0.0003
0	4	0.2350	1.0079 × 10^−5^	0.0000
0	5	0.5675	9.7644 × 10^−5^	0.0001
30	1	0.1403	8.7681 × 10^−3^	0.0088
0	2	0.2620	4.9483 × 10^−4^	0.0005
0	3	0.2255	4.0167 × 10^−4^	0.0004
0	4	0.2311	5.4253 × 10^−7^	0.0000
0	5	0.5563	1.7813 × 10^−6^	0.0000
40	1	0.1668	1.4434 × 10^−2^	0.0144
0	2	0.2684	8.2036 × 10^−4^	0.0008
0	3	0.2397	1.1705 × 10^−3^	0.0012
0	4	0.2344	6.7463 × 10^−6^	0.0000
0	5	0.5527	2.4542 × 10^−5^	0.0000
50	1	0.1894	2.0398 × 10^−2^	0.0204
0	2	0.2707	9.5958 × 10^−4^	0.0010
0	3	0.2500	1.9784 × 10^−3^	0.0020
0	4	0.2409	8.2837 × 10^−5^	0.0001
0	5	0.5510	4.4383 × 10^−5^	0.0000

**Table 2:** OSPA distance and the corresponding parameters for the case of damage occurring on the first floor (El Centro earthquake excitation, 5% noise, autoregressive model, data length = 4000, AR order = 1, OSPA.p = 2, Assignment = 1).

Damage (%)	Floor	Pole	D	OSPA Distance
10	1	0.2554	5.3738 × 10^−4^	0.0005
0	2	0.3177	4.8151 × 10^−5^	0.0000
0	3	0.3236	2.1281 × 10^−7^	0.0000
0	4	0.3339	4.5140 × 10^−5^	0.0000
0	5	0.5858	1.0910 × 10^−5^	0.0000
20	1	0.2676	1.2545 × 10^−3^	0.0013
0	2	0.3277	2.8711 × 10^−4^	0.0003
0	3	0.3179	3.8201 × 10^−5^	0.0000
0	4	0.3393	1.4790 × 10^−4^	0.0001
0	5	0.5793	9.5872 × 10^−5^	0.0001
30	1	0.2915	3.5150 × 10^−3^	0.0035
0	2	0.3269	2.6204 × 10^−4^	0.0003
0	3	0.3161	6.3640 × 10^−5^	0.0001
0	4	0.3361	7.9809 × 10^−5^	0.0001
0	5	0.5799	8.4547 × 10^−5^	0.0001
40	1	0.3138	6.6601 × 10^−3^	0.0067
0	2	0.3248	1.9730 × 10^−4^	0.0002
0	3	0.3114	1.6101 × 10^−4^	0.0002
0	4	0.3395	1.5355 × 10^−4^	0.0002
0	5	0.5748	2.0421 × 10^−4^	0.0002
50	1	0.3302	9.5953 × 10^−3^	0.0096
0	2	0.3243	1.8263 × 10^−4^	0.0002
0	3	0.2909	1.0986 × 10^−3^	0.0011
0	4	0.3411	1.9605 × 10^−4^	0.0002
0	5	0.5530	1.3033 × 10^−3^	0.0013

**Table 3:** Specific physical parameters of the columns.

Column	Section *h* × *b* × *l* (m^3^)	Theoretical Lateral Stiffness (N/m)	State
Type 0	0.003 × 0.030 × 0.24	1.1809 × 10^4^	Undamaged
Type 1	0.003 × 0.014 × 0.24	5.5110 × 10^3^	53.33% Damage
Type 2	0.003 × 0.010 × 0.24	3.9364 × 10^3^	66.67% Damage
Type 3	0.003 × 0.006 × 0.24	2.3619 × 10^3^	80.00% Damage

**Table 4:** OSPA distance and the corresponding parameters for the case of damage occurring on the first floor (sine sweeping-frequency excitation, autoregressive model, data length = 4000, AR order = 1, OSPA.p = 2, Assignment = 1).

Damage (%)	Floor	Pole	D	OSPA Distance
53.33	1	0.8875	1.6746 × 10^−3^	0.0017
0	2	0.8405	6.6544 × 10^−6^	0.0000
0	3	0.8374	1.8927 × 10^−6^	0.0000
0	4	0.8325	9.7249 × 10^−6^	0.0000
0	5	0.9097	8.4550 × 10^−6^	0.0000
66.67	1	0.8992	2.7698 × 10^−3^	0.0028
0	2	0.8478	9.6323 × 10^−5^	0.0001
0	3	0.8408	2.2149 × 10^−5^	0.0000
0	4	0.8368	1.3291 × 10^−6^	0.0000
0	5	0.9121	2.4982 × 10^−7^	0.0000
80.00	1	0.9105	4.0871 × 10^−3^	0.0041
0	2	0.8422	1.8414 × 10^−5^	0.0000
0	3	0.8362	9.6462 × 10^−9^	0.0000
0	4	0.8381	6.3794 × 10^−6^	0.0000
0	5	0.9161	1.2693 × 10^−5^	0.0000

**Table 5:** Modal characteristics of the experimental building model.

Mode	Natural Frequency (Hz)	Damping Ratio (%)
1	2.93	0.17
2	8.91	0.14
3	14.82	0.13
4	20.33	0.08
5	24.40	0.05

**Table 6:** OSPA distance and the corresponding parameters for the case of removing braces on the first floor (white noises excitation, autoregressive model, data length = 4000, AR order = 2, OSPA.p = 2).

Damage	Floor	Pole	D		Assignment		OSPA Distance
**Removing**	**1**	0.6667 ± 0.5754i	0.0408	1.6938	1	0	0.0408
braces			1.6938	0.0408	0	1	
0	2	0.5520 ± 0.6307i	0.0065	1.7785	1	0	0.0065
			1.7785	0.0065	0	1	
0	3	0.5710 ± 0.6322i	0.0078	1.7823	1	0	0.0078
			1.7823	0.0078	0	1	
0	4	0.5716 ± 0.6117i	0.0053	1.6293	1	0	0.0053
			1.6293	0.0053	0	1	
0	5	0.7160 ± 0.5228i	0.0033	1.1546	1	0	0.0033
			1.1546	0.0033	0	1	

**Table 7:** OSPA distance and the corresponding parameters for the case of removing columns on the first floor (white noises excitation, autoregressive model, data length = 4000, AR order = 2, OSPA.p = 2).

Damage	Floor	Pole	D		Assignment		OSPA Distance
**Removing**	**1**	0.7890 ± 0.5326i	0.0051	1.2227	1	0	0.0051
columns			1.2227	0.0051	0	1	
0	2	0.7231 ± 0.5873i	0.0004	1.3890	1	0	0.0004
			1.3890	0.0004	0	1	
0	3	0.7202 ± 0.5862i	0.0002	1.3895	1	0	0.0002
			1.3895	0.0002	0	1	
0	4	0.7260 ± 0.5780i	0.0002	1.3496	1	0	0.0003
			1.3496	0.0002	0	1	
0	5	0.8310 ± 0.4280i	0.0007	0.7145	1	0	0.0007
			0.7145	0.0007	0	1	
